# Bundesweites Belastungsmodell für Intensivstationen durch COVID-19

**DOI:** 10.1007/s00063-021-00791-7

**Published:** 2021-02-03

**Authors:** A. Schuppert, S. Theisen, P. Fränkel, S. Weber-Carstens, C. Karagiannidis

**Affiliations:** 1 Institut für Computational Biomedicine, Universitätsklinikum Aachen, RWTH Aachen, Pauwelsstraße 19, 52074 Aachen, Deutschland; 2 Vorstandsstab Universitätsklinikum Aachen, RWTH Aachen, Aachen, Deutschland; 3 Klinik für Anästhesiologie und operative Intensivmedizin (CCM, CVK), Charité – Universitätsmedizin Berlin, Berlin, Deutschland; 4 ARDS und ECMO Zentrum Köln-Merheim, Kliniken der Stadt Köln, Universität Witten/Herdecke, Köln, Deutschland

**Keywords:** ARDS, ICU, Modell, Szenario, Simulation, Acute respiratory distress syndrome, ICU, Model, Scenario, Simulation

## Abstract

**Hintergrund:**

Prognosemodelle zur Intensivbelegung mit COVID-19-Patienten sind in der aktuellen Pandemie wichtig zur strategischen Planung der Patientenallokation und Vermeidung regionaler Überlastung. Sie werden oft vollständig an retrospektiven Infektions- und Belegungsdaten trainiert, wodurch die Prognoseunsicherheit exponentiell mit dem Prognosehorizont anwachsen kann.

**Methodik:**

Wir schlagen einen alternativen Modellansatz vor, bei dem das Modell weitgehend unabhängig von den zu simulierenden Belegungsdaten erstellt wird. Die Verteilung der Bettenbelegungen für Patientenkohorten wird direkt aus Belegungsdaten aus „Sentinel-Kliniken“ berechnet. Durch Kopplung mit Infektionsszenarien wird der Prognosefehler durch den Fehler der Infektionsdynamikszenarien beschränkt. Das Modell erlaubt eine systematische Simulation von beliebigen Infektionsszenarien, die Berechnung von Korridoren für die Bettenauslastung sowie Sensitivitätsanalysen im Hinblick auf Schutzmaßnahmen.

**Ergebnisse:**

Das Modell wurde anhand von Klinikdaten und durch Anpassung von nur 2 Parametern an die Daten in der Städteregion Aachen und Deutschland gesamt vorgenommen.

Am Beispiel der Simulation der jeweiligen Bettenbelegungen für das Bundesgebiet wird das Belastungsmodell zur Berechnung von Belegungskorridoren demonstriert. Die Belegungskorridore bilden Schranken für die Bettenbelegungen für den Fall, dass die Infektionszahlen spezifische Grenzwerte nicht überschreiten. Darüber hinaus werden Lockdownszenarien simuliert, die sich an retrospektiven Ereignissen orientieren.

**Diskussion:**

Unser Modell zeigt, dass eine deutliche Reduktion der Prognoseunsicherheit in Auslastungsprognosen durch gezielte Kombination von Daten aus unterschiedlichen Quellen möglich ist. Es erlaubt eine beliebige Kombination mit Modellen und Szenarien zur Infektionsdynamik und kann damit sowohl zur Belastungsprognose als auch für Sensitivitätsanalysen für zu erwartende neuartige Spreading- und Lockdownszenarien eingesetzt werden.

**Zusatzmaterial online:**

Die Onlineversion dieses Beitrags (10.1007/s00063-021-00791-7) enthält die Simulation der Prognosekorridore der Intensivbettenbelegung für die Bundesländer. Beitrag und Zusatzmaterial stehen Ihnen auf www.springermedizin.de zur Verfügung. Bitte geben Sie dort den Beitragstitel in die Suche ein, das Zusatzmaterial finden Sie beim Beitrag unter „Ergänzende Inhalte“.

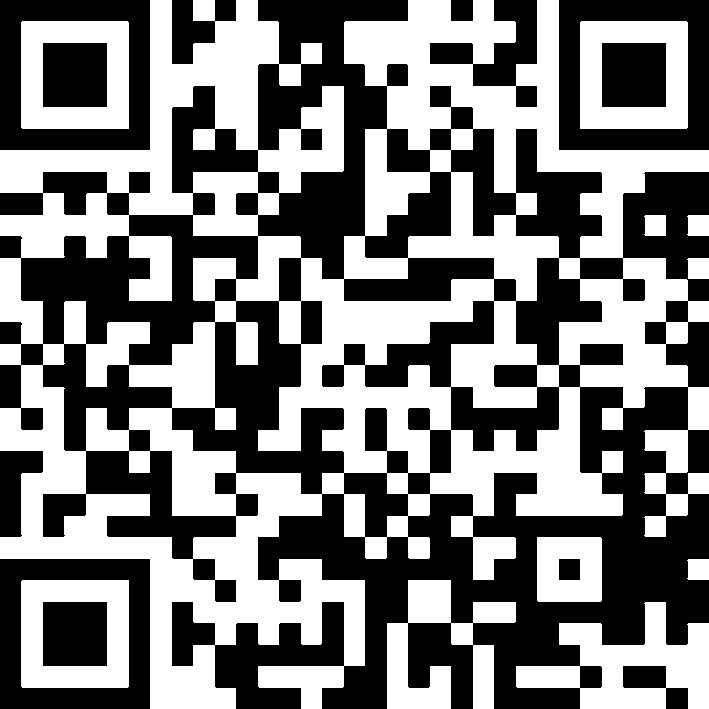

## Abstract (en)

**Background:**

Forecasting models for intensive care occupancy of coronavirus disease 2019 (COVID-19) patients are important in the current pandemic for strategic planning of patient allocation and avoidance of regional overcrowding. They are often trained entirely on retrospective infection and occupancy data, which can cause forecast uncertainty to grow exponentially with the forecast horizon.

**Methodology:**

We propose an alternative modeling approach in which the model is created largely independent of the occupancy data being simulated. The distribution of bed occupancies for patient cohorts is calculated directly from occupancy data from “sentinel clinics”. By coupling with infection scenarios, the prediction error is constrained by the error of the infection dynamics scenarios. The model allows systematic simulation of arbitrary infection scenarios, calculation of bed occupancy corridors, and sensitivity analyses with respect to protective measures.

**Results:**

The model was based on hospital data and by adjusting only two parameters of data in the Aachen city region and Germany as a whole. Using the example of the simulation of the respective bed occupancy rates for Germany as a whole, the loading model for the calculation of occupancy corridors is demonstrated. The occupancy corridors form barriers for bed occupancy in the event that infection rates do not exceed specific thresholds. In addition, lockdown scenarios are simulated based on retrospective events.

**Discussion:**

Our model demonstrates that a significant reduction in forecast uncertainty in occupancy forecasts is possible by selectively combining data from different sources. It allows arbitrary combination with infection dynamics models and scenarios, and thus can be used both for load forecasting and for sensitivity analyses for expected novel spreading and lockdown scenarios.

Die SARS-CoV-2-Pandemie hat im Rahmen der ersten COVID-19-Welle im Frühjahr 2020 zu einer Spitzenbelastung der Intensivstationen mit mehr als 3500 COVID-19-Patienten im April 2020 geführt, die durch den ersten Lockdown bis Anfang Oktober 2020 auf niedriges Niveau zurückgeführt werden konnten. Der im Oktober einsetzende starke Anstieg der Infektionen führte zu einer zunehmenden Belastung der Intensivmedizin. Trotz der hohen Intensivbettenzahl drohen die Ressourcen im Winter 2020/2021 vollständig ausgelastet zu werden [1]. Im Hinblick auf eine Kapazitätssteuerung im deutschen Gesundheitswesen ist eine Planung mithilfe von Prognosemodellen zum weiteren Verlauf der COVID-19-Infektionsdynamik essenziell. Hierbei spielt die Latenzzeit bis zum Auftreten der respiratorischen Insuffizienz und Intensivpflichtigkeit eine besondere Rolle.

Integrierte Prognosemodelle für den Verlauf der Covid19-Infektion und die damit einhergehenden Belastungen für die Krankenversorgung auf Basis von SEIR(Susceptible – Exposed – Infected – Removed – Modelle)-Modellen [2–6] modellieren sowohl die Infektionsdynamik als auch die daraus resultierenden Belastungen in einem einheitlichen Modellsystem. Hierbei wird die Bevölkerungspopulation in Kompartimente eingeteilt mit jeweils homogener intrinsischer stochastischer Dynamik. Die Belegungen von Normalstationen, Intensivstationen sowie der Beatmungsplätze können jeweils als eigenständige Kompartimente integriert oder als separates Modell dargestellt werden. Das mittlere Verhalten des Systems wird durch gewöhnliche Differenzialgleichungen quantifiziert, deren Parametrisierung durch Anpassung an retrospektive Daten zur Infektionsdynamik und der Bettenbelegungssituation vorgenommen wird.

Modelle vom SEIR-Typ weisen in einer Pandemiephase weit unterhalb der Herdenimmunität eine intrinsische exponentielle Instabilität des Lösungsverhaltens auf. Hierdurch resultieren Unsicherheiten im Gesamtmodell, die tendenziell exponentiell mit dem Prognosehorizont wachsen. Außerdem ist die Verknüpfung der Parametrisierung durch veränderte politische Rahmenbedingungen nicht explizit integriert, sondern muss aus Daten retrospektiv geschätzt werden, wodurch ein Zeitverzug resultiert. Netzwerkbasierte Modelle [7], die das Kontaktverhalten der Bevölkerung explizit abbilden, sind in Deutschland wegen der unzulänglichen Datenlage nur sehr schwer zu parametrisieren.

Daher schlagen wir ein Belastungsmodell vor, bei dem die Dynamik des Infektionsgeschehens explizit entkoppelt wird von der Modellierung der Bettenbelegungen und nur die zu erwartende Dynamik der Bettenbelegung bei vorgegebener Infektionsdynamik simuliert wird. Das Belastungsmodell ist dabei so ausgelegt, dass es die reale Verteilung der Bettenbelegungsdauern explizit erfasst und so auch atypische Verteilungen simulieren kann. Das Modell wird dabei bis auf wenige Parameter nicht mithilfe einer gegebenen Infektionsdynamik und zugehörigen Bettenbelegungen parametrisiert, sondern mithilfe von separat erhobenen Belegungsdaten aus „Sentinel“-Kliniken. Daher ist die Identifizierung des Belastungsmodells numerisch robust, d. h., kleine Veränderungen in den Parametern und den Infektionsdynamiken können qua Design keine zeitlich exponentiell anwachsenden Auswirkungen auf die Bettenbelegungen haben. Durch unser Modelldesign wird numerische Robustheit und Prognosesicherheit gewonnen, dafür verlangt unser Ansatz eine geeignete Auswahl von „Sentinel“-Kliniken mit ausreichender Verfügbarkeit von Daten. Die vorliegende Arbeit soll zeigen, dass schon mit Daten von wenigen „Sentinel“-Kliniken mit hohen Belegungszahlen eine gute Modellqualität mit großer Generalisierbarkeit auf Länder- und Bundesebene erreicht werden kann.

## Mathematische Struktur des Belastungsmodells

Das Belastungsmodell geht von einer gegebenen Infektionsdynamik, u_1_(t), … u_m_(t), aus mit u_i_(t) als der täglichen Infektionszahl der i‑ten Altersgruppe im zu simulierenden Gebiet. u_i_(t) können aus Datenquellen, Prognosen oder Simulationen von Szenarien gewonnen werden.

Der Kern des Modells ist die Quantifizierung des erwarteten zukünftigen Bettenbedarfs der Kategorie k (Hospitalisierung allgemein, Intensivbett, Intensivbett mit Beatmung) zum Zeitpunkt t für einen zum Zeitpunkt t′ infizierten Patienten der Altersgruppe i quantifiziert durch die Belastungsfunktion B(t-t′,k,i), B(t-t′,k,i) = 0 für t < t′.

Die Gesamtbelastung GB(t) des Gesundheitssystems der Kategorie k im zu simulierenden Gebiet zum Zeitpunkt t ist dann eine Faltung der Belastungsfunktion B(t-t′,k,i) mit den Infektionszahlen u_k_(t):

1 $$GB_{k}\left(t\right)=\sum _{i}\int _{0}^{t}B\left(t-t',k,i\right)\cdot u_{i}\left(t'\right)dt'.$$

Abweichungen δu_i_(t′) der Infektionsdynamik vom angenommenen Szenario u_i_(t) resultieren in einer Abweichung δGB(t):

$$\delta GB_{k}\left(t\right)=\sum _{i}\int _{0}^{t}B\left(t-t\mathrm{'},k,i\right)\cdot \delta u_{i}\left(t\mathrm{'}\right)dt\mathrm{'}.$$

Die Hölder-Ungleichung garantiert, dass die resultierende Abweichung stets innerhalb eines Korridors von der Breite

1a $$|\updelta GB_{k}\left(t\right)|\leq \sum _{i}\int _{0}^{t}B\left(t-t\mathrm{'},k,i\right)dt\mathrm{'}\cdot \max \left(\left| \delta u_{i}\left(t\mathrm{'}\right)\right| \right)$$

liegen muss. Das Modell erlaubt daher die Berechnung von Korridoren für maximale Ausschläge der Belastung in Abhängigkeit der Infektionsdynamik unabhängig von deren konkreter Realisierung.

Die Berechnung der Belastungsfunktion B(t-t′,k,i) aus gemessenen Belegungs- und Infektionsdaten mithilfe von Maximum-Likelihood-Verfahren aus der Modellgleichung Gl. 1 ist jedoch instabil. Daher schätzen wir die Belastungsfunktion B(t-t′,k,i) direkt aus Belegungsdaten von „Sentinel“-Krankenhäusern.

Da weder der Zeitpunkt der Infektion t′ noch der genaue Zeitpunkt der Aufnahme auf die Intensivstation sowie der Beginn der Beatmung bekannt ist, muss im Modell für jede Bettenkategorie ein unbekannter Zeitverzug aus den Belegungsdaten für jede Bettenkategorie einzeln geschätzt werden. Hierfür wird die Belastungsfunktion B(t-t′,k,i) approximiert werden durch

2 $$B\left(t-t\mathrm{'},k,i\right)=R\left(k,i\right)\cdot \int S\left(t_{\text{aufnahme}}-t\mathrm{'},k\right)\cdot B'\left(t_{\mathrm{LoS}},k,i\right)dt\mathrm{'},$$

wobei B′(t_LoS_,k,i) die Verteilung der Aufenthaltsdauer der Patienten in Bettenkategorie k ist. S(t_aufnahme_ − t′,k) ist die Wahrscheinlichkeitsverteilung für den Zeitverzug zwischen Einweisung und Infektion und R(k,i) die Wahrscheinlichkeit für einen Bettenbedarf der Kategorie k für einen infizierten Patienten der Altersgruppe i.

S wurde normalverteilt um t_e_(k) mit einer Spannbreite von 7 Tagen angenommen. Da zwischen Infektion, Erfassung der Diagnose und Einweisung ein unbekannter Zeitverzug besteht, der außerdem zeitlich leicht schwanken kann, wird t_e_(k) aus Belegungs- und Infektionsdaten mithilfe der Gl. 1 direkt geschätzt.

Das Risiko R(k,i) wird approximiert als Produkt aus dem relativen Risiko der Altersgruppen R_rel_(k,i) multipliziert mit einem von der Kategorie k abhängigen Gesamtrisikofaktor R_g_(k):

$$R\left(k,i\right)=R_{g}\left(k\right)R_{\mathrm{rel}}\left(k,i\right).$$

R_rel_(k,i) wird aus der Verteilung der Altersgruppen in den „Sentinel“-Kliniken und der Altersverteilung der Infektionsdynamiken geschätzt. Das absolute Risiko R_g_(k) für jede Bettenkategorie wird mit Gl. 1 aus der Infektions- und Bettenbelegungdynamik geschätzt.

Für die Schätzung von R_g_(k) und t_e_(k) wird ein „least square fit“ mit Kreuzvalidierung (80 % Trainingsdaten, 20 % Testdaten) verwendet, die t_e_(k) und R_g_(k) werden als Erwartungswert der in jedem Kreuzvalidierungslauf gefitteten Parameter berechnet. Als Modellunsicherheit wird der Fehler im Testset angenommen.

## Daten

In der vorliegenden Modellversion wurden zur Modellidentifizierung Daten aus den folgenden Quellen verwendet:

### Infektionsdynamik

- Dashboard Robert Koch Institut (RKI) [8]. Es wurden die verfügbaren 6 Altersstufen zur Stratifizierung verwendet, eine weitere Stratifizierung nach Geschlecht wurde nicht vorgenommen
- tägliche Infektionszahlen (RKI) [9]: Für die jeweils letzten 14 Tage werden die relativen Fallzahlen der jeweiligen Altersgruppen berechnet und mithilfe der gemeldeten Infektionszahlen (ohne Altersstratifizierung) kalibriert, ansonsten werden die Daten direkt verwendet.
  Zur Kompensation der 7‑Tage-Periodizität der Infektionsreportings wurden die tagesaktuellen Daten geglättet. Hierzu wurden die Daten des jeweiligen Tags über die letzten 5 Wochen mit einem kubischen „spline“ interpoliert und jeder Tag durch den Median der resultierenden 7 „splines“ approximiert.
  Testintensität (RKI) [10]: Die Infektionszahlen der ersten Welle wurden entsprechend der in den RKI-Situationsberichten gemeldeten Testintensitäten korrigiert. Die Korrektur spielt keine Rolle für die Daten der zweiten Welle sowie für die Szenarioprognosen

### Bettenbelegungen

- Die Parameterschätzung des lokalen Modells für die Städteregion Aachen wurde anhand der Daten IG NRW durchgeführt
- Für Deutschland wurden Daten des DIVI-Intensivregisters [11] verwendet. Die Simulationen für die einzelnen Bundesländer wurden mit dem an den Daten für Deutschland gesamt parametrisierten Modell ohne länderspezifische Parameteranpassung durchgeführt.

### Belegungsdauern

- Die Belastungsfunktionen B′(t_LoS_,k,i) wurden mithilfe von anonymisierten Patientendaten der Uniklinik RWTH Aachen (*N* = 451) sowie des Krankenhauses Köln-Merheim (Klinikum der Universität Witten-Herdecke; *N* = 66 Intensivpatienten) berechnet. Es werden nur Patienten eingeschlossen, die die Behandlung zum Zeitpunkt der Modellierung abgeschlossen haben und ab Mai 2020 aufgenommen wurden.

## Implementierung und Parametrisierungsstrategie

Alle Berechnungen wurden mithilfe von MatLab, Version R2020a, The Mathworks Inc, Natick, MA, USA, Basisversion und Toolbox „Statistics and Machine Learning“, durchgeführt.

Die Schätzungen der Belastungsfunktionen B′(t_LoS_,k,i) sowie der Risikofunktionen wurden mit 100-fach-Bootstrapping mit jeweils 80 % der Daten durchgeführt.

Bei der Berechnung der Parameter t_e_(k) und R_g_(k) für die Städteregion wurden die gemeldeten Bettenbelegungen um die Zuweisungen von außerhalb der Städteregion für das UK Aachen als Hauptträger der Zuweisungen reduziert. Die Parameter für Deutschland wurden aus den DIVI-Intensivregisterdaten ohne Korrekturen geschätzt. Es wurde 20-fache Kreuzvalidierung mit jeweils 80/20-Splitting in Trainings- und Testdatensatz verwendet. Als Fehlerfunktion wurde für jede Kategorie der mit der absoluten Belegungszahl gewichtete quadratische relative Fehler sowohl im Training- als auch im Testdatensatz verwendet.

## Modellparametrisierung

Alle Belegungsdaten wurden auf die Altersgruppen des RKI-Dashboard bezogen. Die Verteilung der Patienten des UK Aachen seit Mai 2020 mit abgeschlossener Behandlung ist in Tab. 1 dargestellt.

**Table 1:** 

*Altersgruppe*	1: 0–4	2: 5–14	3: 15–34	4: 35–59	5: 60–80	6: 80+
*Hospitalisierung*	3	6	19	65	82	48
*ICU*	0	1	5	27	30	10
*ICU & MV (mechanische Beatmung)*	0	1	2	22	25	6

**Figure 1:**
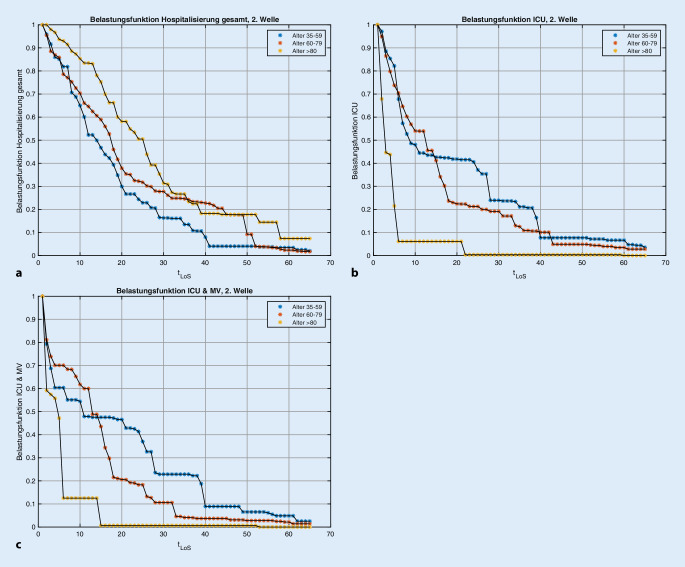

Die daraus berechneten Belastungsfunktionen B′ für die Altersgruppen 4–6 sind in Abb. 1a–c dargestellt. Die zusätzlichen Daten aus Köln-Merheim ergeben keine signifikante Änderung.

**Figure 2:**
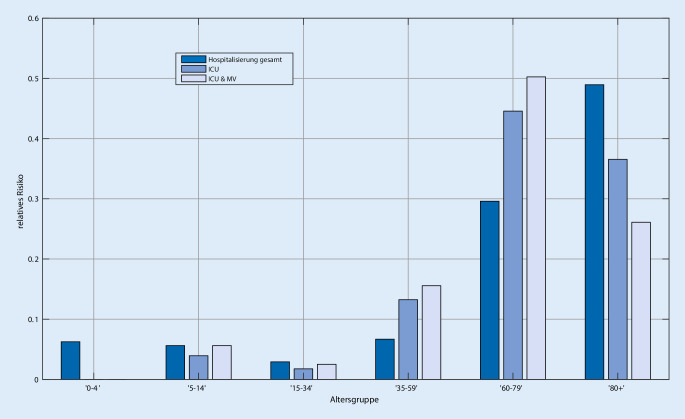

Das relative Risiko R_rel_ (k,i), geschätzt aus den Belegungsdaten des Universitätsklinikums Aachen ist in Abb. 2 dargestellt.

Wie erwartet steigt das Risiko innerhalb jeder Bettenkategorie mit dem Alter deutlich an, wobei in der Intensiv- und Beatmungskategorie der Abfall in der Altersgruppe ≥ 80 Jahre auffällt. Dieser kann jedoch auf die Patientenverfügungen zurückgeführt werden.

Mithilfe der in Abb. 1 und 2 angegebenen Verteilungen für die Parametrisierungen werden die verbleibenden Parameter t_e_(k) und R_g_(k) anhand eines Maximum-Likelihood-Schätzers mit Kreuzvalidierung identifiziert. Der relative gewichtete Fehler für die Städteregion Aachen zum Zeitpunkt 16.12.2020 liegt zwischen 10–20 % (Tab. 2):

**Table 2:** 

	Relativer Fehler Training [%] (Region Aachen 16.12.2020)	Relativer Fehler Testset [%] (Region Aachen 16.12.2020)
*Hospitalisierung gesamt*	20 (10–20)	15
*ICU*	13 (10–17)	11
*ICU & MV*	16 (10–17)	11

Der Modellfehler für die Städteregion Aachen wird stark geprägt durch lokale Spreading-Events in Alters- und Pflegeheimen. Durch die Glättung der Wochenperioden in den Meldezahlen kann das Modell nur mit einem Zeitverzug von mindestens einer Woche auf sprunghafte Ereignisse reagieren, die besonders bei der Simulation kleinerer Regionen einen deutlichen Beitrag zur Infektionsdynamik liefern. Hiervon sind besonders die Simulationen der Hospitalisierung gesamt betroffen, die ICU- und ICU-MV-Belegungen zeigen eine niedrigere dynamische Varianz.

Für die Simulation der Bettenbelegungen in Deutschland und den Bundesländern wurden die Parameter t_e_ und R_g_ zusätzlich mithilfe der DIVI-Intensivregisterdaten für Deutschland gesamt geschätzt. Hier ergab sich ein Modellierungsfehler im Bereich 5 % für die ICU-Bettenbelegung und 7 % für die ICU-MV-Belegung.

Die Simulationen für die Bundesländer wurden ohne weitere Parameteranpassungen durchgeführt.

## Szenariosimulationen

Das parametrisierte Belastungsmodell wurde eingesetzt zur Simulation verschiedener Szenarien.

### Szenario 1: Simulation von Belegungskorridoren

Als Basisszenario wird der aktuelle 7‑Tage-Infektionswert des jeweiligen Gebiets in den 6 Altersgruppen angenommen und die Basisbelegung für die 3 Belegungskategorien berechnet. Dann wird eine sprunghafte altersproportionale Erhöhung/Reduktion der Infektionszahlen ab dem jeweiligen Stichtag um 10, 25 und 50 % angenommen und die resultierenden Belegungszahlen simuliert. Dadurch ergeben sich jeweils obere und untere Schranken für die Belegungszahlen, die sich wegen der Trägheit des individuellen Infektionsverlaufs dynamisch im Verlauf von wenigen Wochen an die jeweiligen stationären Werte anpassen. Diese Korridore bilden obere und untere Schranken für die Belastungen, sodass unabhängig von der realen Infektionsdynamik diese simulierten Belastungsgrenzen nicht über- oder unterschritten werden können, solange die Infektionsdynamik den jeweiligen Schwellwert nicht überschreitet.

**Figure 3:**
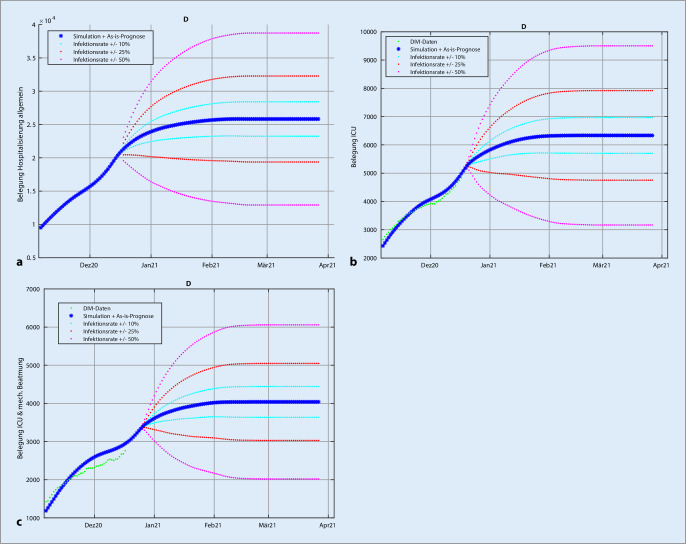

Die jeweiligen Simulationen für Deutschland sind Stand 22.12.2020 exemplarisch in Abb. 3 dargestellt. Die Fehlerschranken sind jeweils die oben angegebenen Modellfehler. Als Basiswert für die mittlere 7‑Tage-Inzidenz in der Woche vor dem 22.12.2020 wurde 24.800 Infektionen pro Tag berechnet. Die Belegungsdaten für eine konstante Inzidenz von 24.800/Tag ab dem 22.12.2020 werden von der blauen Kurve beschrieben. Die jeweiligen Inzidenzwerte für die Korridore sind in der Legende angegeben. Die Korridore können wie im folgenden Beispiel interpretiert werden: Bei einer täglichen Inzidenz über einen längeren Zeitraum zwischen 12.400 und 18.600 werden sich die Belegungen zwischen der untersten und der unteren roten Kurve einpendeln. Hiermit können die Belegungskorridore zur Abschätzung der Belastungen beim Stopp eines Lockdowns eingesetzt werden. Entsprechende Daten für die ICU-Belegungen der Länder sind im elektronischen Zusatzmaterial online dargestellt.

### Szenario 2: Simulation von Lockdownmaßnahmen

Im Gegensatz zur Simulation der Prognosekorridore, bei der keine Modellierung der Infektionsdynamik notwendig war, ist die Simulation von dynamischen Szenarien mit einem zusätzlichen Fehler für die Annahmen des Szenarios versehen. Die Infektionsdynamik eines Szenarios kann epidemiologischen Modellen oder datengetrieben aus Analogien mit früheren/ausländischen Lockdowns geschätzt werden. Die Szenariosimulationen sind weniger als quantitative Prognose geeignet, sondern können primär zur Durchführung von Sensitivitätsstudien als auch zur Schätzung von Ober- und Untergrenzen für zu erreichenden Steuergrößen dienen.

Als Beispiel sei hier eine Simulation mit einem Lockdownszenario demonstriert, das analog zur Dynamik des Herbstlockdowns in Frankreich simuliert wurde. Hierzu wurde angenommen, dass sich bis zur Aktivierung des Lockdowns die aktuelle Infektionsdynamik in allen Altersgruppen unverändert fortsetzt. Aus den französischen Infektionsdaten [12] wurde der niedrigste durch den Lockdown in Frankreich erreichte R‑Wert berechnet. Als Dynamik des lockdowninduzierten Übergangs von hohem zu niedrigem Ziel-R-Wert wurde eine exponentielle Dynamik angenommen, deren Parametrisierung aus den Daten des November-Lockdowns aus Deutschland geschätzt wurde. Hieraus wurden die resultierenden Infektionszahlen u_lockdown_(t) als Lösung einer dynamischen Folge simuliert und mithilfe des parametrisierten Modells die resultierenden erwarteten Bettenbelegungen in jeder Kategorie simuliert.

**Figure 4:**
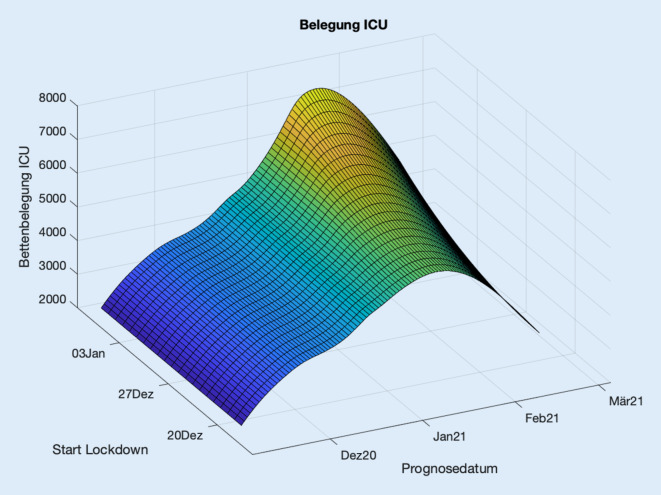

Als Beispiel können die Intensivbettenbelegungen für den Start des Lockdownszenarios ab dem 16.12. simuliert werden (Abb. 4).

**Figure 5:**
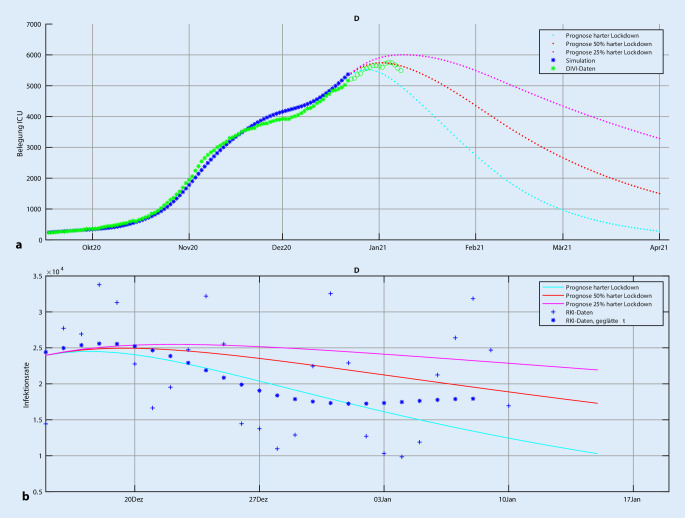

Ein weiteres Beispiel ist die Simulation der Konsequenzen von „weichen“ vs. „harten“ Lockdownszenarien am Beispiel des zweiten Lockdowns in Österreich. Es wird dabei der Start am 16.12. simuliert mit Parametrisierung des zweiten Lockdowns in Österreich sowie 50 und 25 % Effizienz (Abb. 5). Alle simulierten Lockdownszenarien führen zu einem R‑Wert kleiner 1 und damit zu einem mehr oder weniger schnellen Abklingen der Infektionsdynamik. Bis zum 24.12. ist eine Diskriminierung zwischen den Lockdownszenarien nicht sicher erreichbar. Der beobachtete Anstieg der geglätteten Daten sind stark beeinträchtig durch den erheblichen Zeitversatz zwischen Infektion und Reporting.

## Resümee

Das vorgestellte Belastungsmodell erlaubt die Simulation der Bettenbelegung in den Kategorien Krankenhaus, Intensivstation und Intensivstation mit mechanischer Beatmung für beliebige Szenarien der Infektionsdynamik. Der Schwerpunkt des Modells ist die numerisch robuste Simulation der resultierenden Bettenbelegungen ohne exponentielles Wachstum der Unsicherheiten. Dies wird durch die weitgehende Schätzung der Modellparameter aus separaten Daten von „Sentinel“-Klinken erreicht. Es erlaubt eine numerisch stabile Simulation von Infektionsszenarien und eignet sich als Simulator bei Optimierungsfragestellungen. Es ist daher nicht als Ersatz, sondern als Ergänzung zu den etablierten epidemiologischen Modellen der Infektionsdynamik zu sehen.

Trotz der numerischen Stabilität des Modellansatzes bleiben für eine breite Nutzung, insbesondere für die lokale Simulation, offene Herausforderungen zu lösen: Die Belastungsfunktion wurde bisher auf Basis von Daten der Uniklinik der RWTH Aachen und des Krankenhauses Köln-Merheim berechnet. Auch wenn die Fallzahlen ausreichend erscheinen und beide Kliniken als repräsentativ für entsprechende Maximalversorger in Deutschland für COVID19-Patienten gelten können, sind für eine robuste Prognose insbesondere in ländlich geprägten Gebieten weitere repräsentative Daten erforderlich.

Eine weitere Unsicherheit liegt in der Konstanz der Datenqualität, insbesondere der Infektionsdaten, die durch Meldeverzug sowie limitierte Laborkapazitäten einen variablen zeitlichen Verzug haben können.

Des Weiteren enthalten die Simulationen durch die wochenperiodischen Glättungen einen Zeitverzug, der besonders bei lokalen Simulationen bei lokalen Outbreak-Events zu zeitverzögerten Prognosen führen kann.

Insgesamt konnten wir zeigen, dass eine Modellierungsstrategie, die die Modellierung der Infektionsdynamik von der Modellierung der resultierenden Bettenbelegungen numerisch und datentechnisch entkoppelt, zu einer deutlichen Reduktion der Prognosefehler beitragen kann und für Szenariosimulationen geeignet ist. Eine systematische Weiterentwicklung in Richtung einer breiten, lokalen Nutzung, auch für zukünftige Infektionslagen, erfordert jedoch die systematische Verfügbarkeit entsprechender Versorgungsdaten aus einem breiteren Panel von „Sentinel“-Kliniken und unterstreicht damit die Bedeutung einer landesweiten Koordination der Datenverfügbarkeit für Forschung und Versorgung.

## Supplementary Information




