# Supplementary figures and images for: Nationwide exposure model for COVID-19 intensive care unit admission
Source: Med Klin Intensivmed Notfmed. 2021 Feb 3;117(3):218–26. [Article in German] doi: 10.1007/s00063-021-00791-7 (PMC7856858; doi:10.1007/s00063-021-00791-7)

## Simulation der Prognosekorridore der Intensivbettenbelegung für die Bundesländer

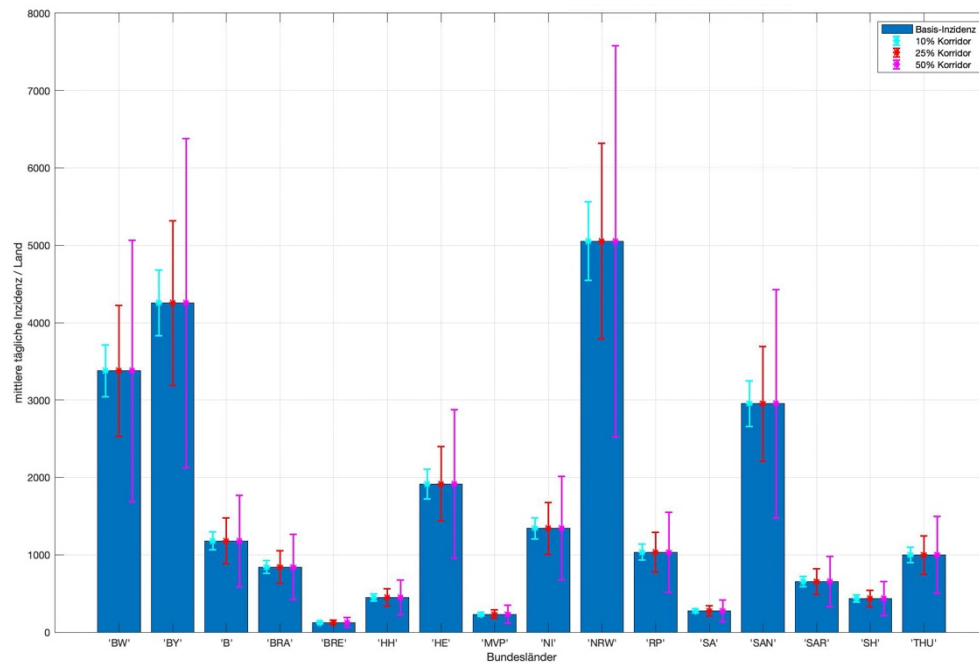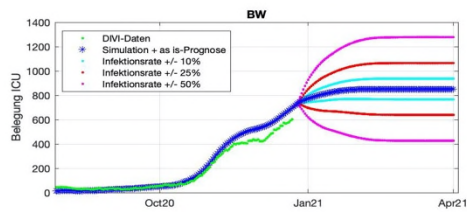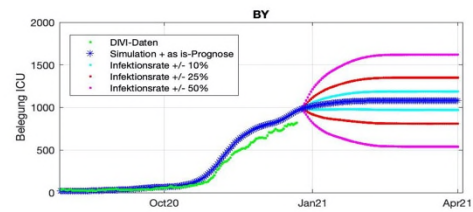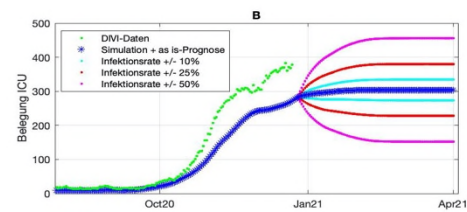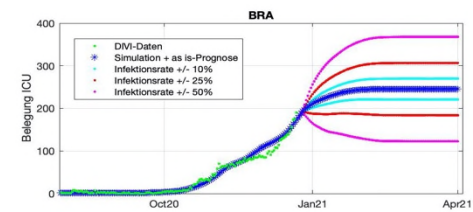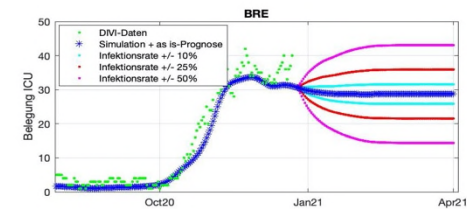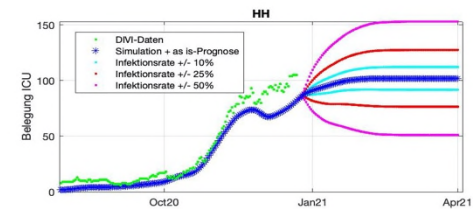

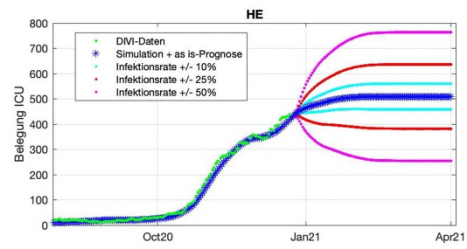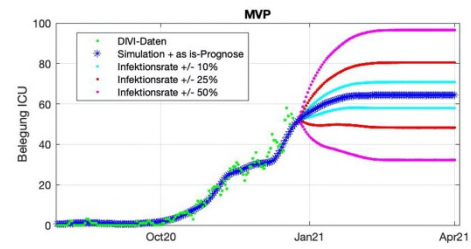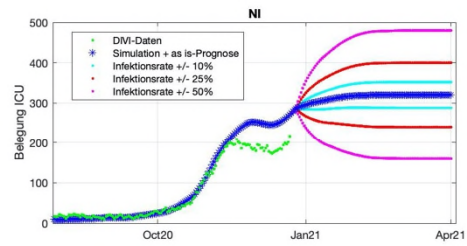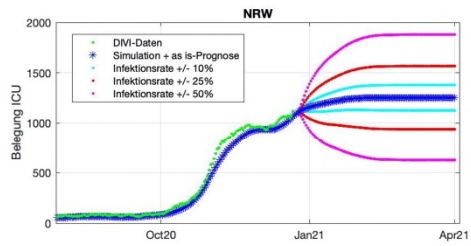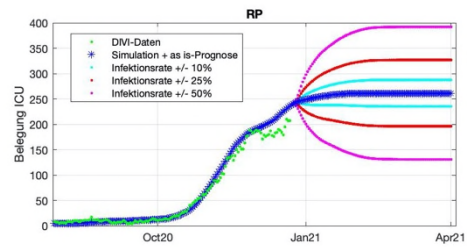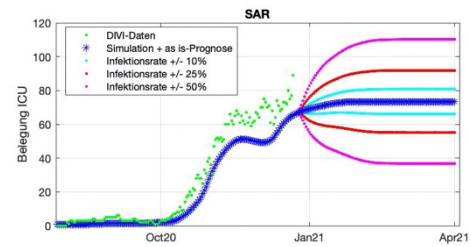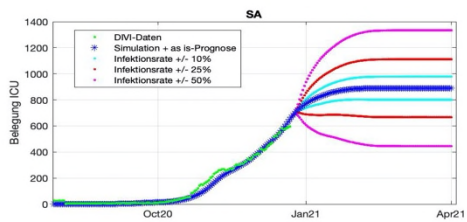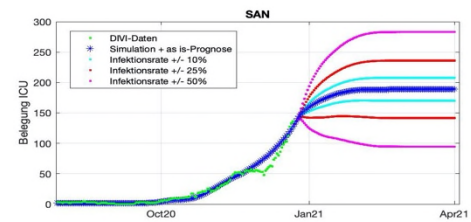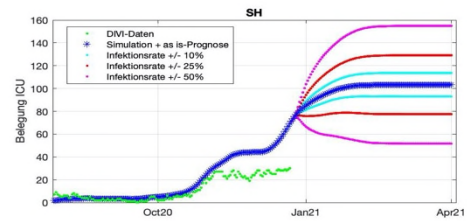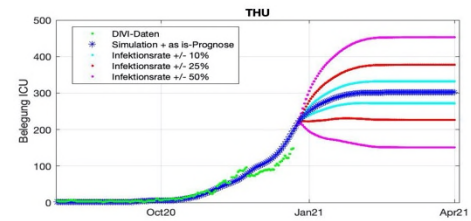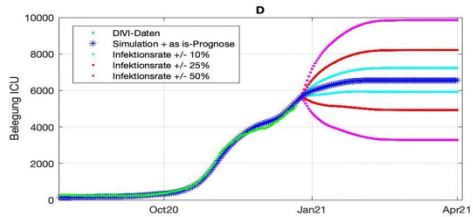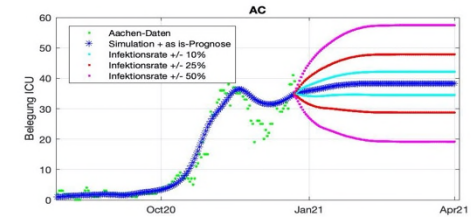

Supplement: Supplementary file 1 [file 63_2021_791_MOESM1_ESM.pdf]
